# Supplementary material for: Long-term cardiovascular disease outcomes in non-hospitalized medicare beneficiaries diagnosed with COVID-19: Population-based matched cohort study
Source: PLoS One. 2024 May 14;19(5):e0302593. doi: 10.1371/journal.pone.0302593 (PMC11093379; doi:10.1371/journal.pone.0302593)
Supplement: S3 Fig — (DOCX) [file pone.0302593.s003.docx]

**S3 Fig. Cause-specific time-varying hazard ratios (95% CI) for risk of death, hospitalization, incident CVD and stroke associated with non-hospitalized COVID-19 by race/ethnicity, Medicare 2020–2021 Matched Cohort**


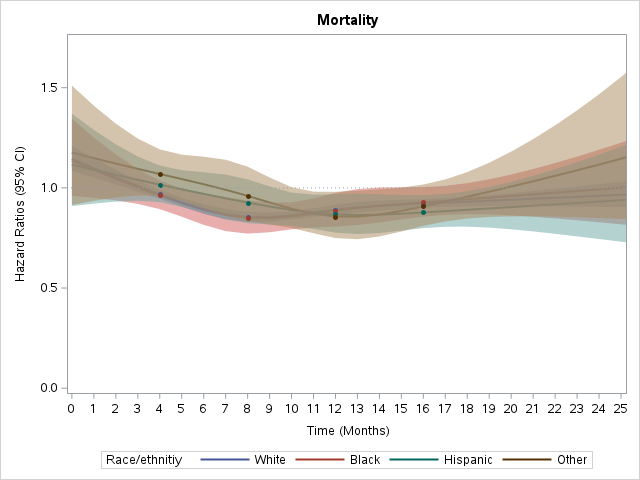


P=1.0 for interaction ^a^


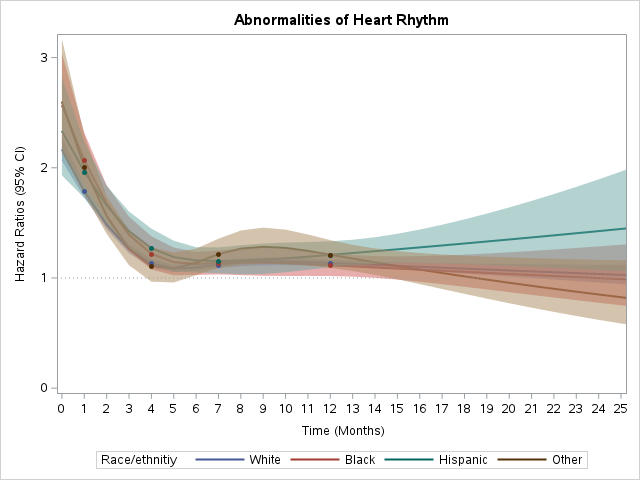


P<0.001 for interaction ^a^


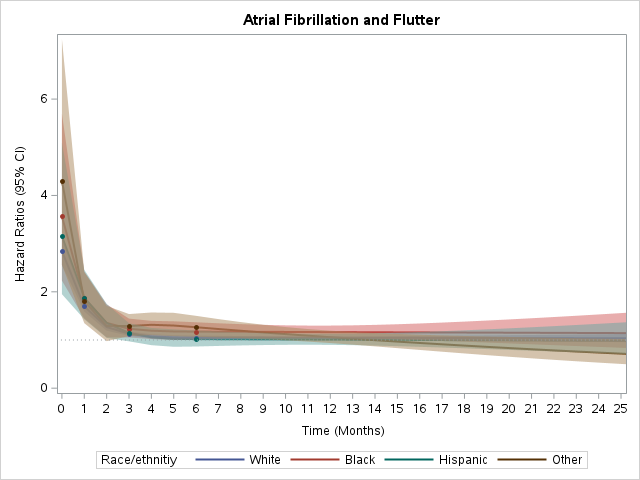


P=0.483 for interaction ^a^


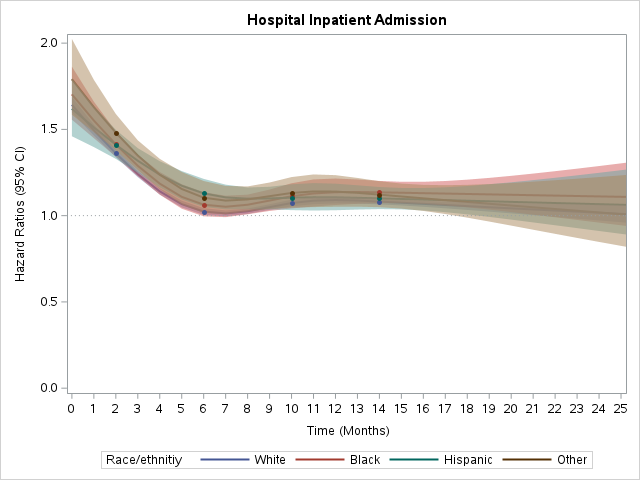


P<0.001 for interaction ^a^


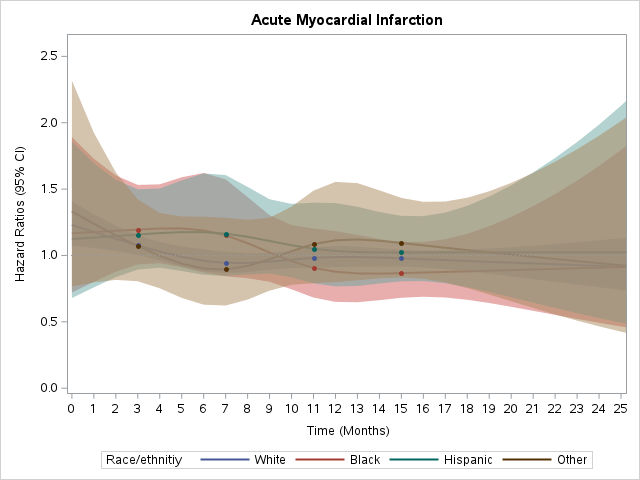


P=1.0 for interaction ^a^


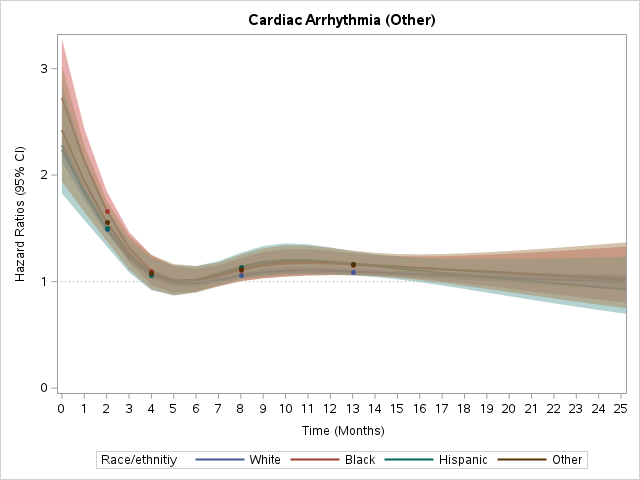


P<0.001 for interaction ^a^


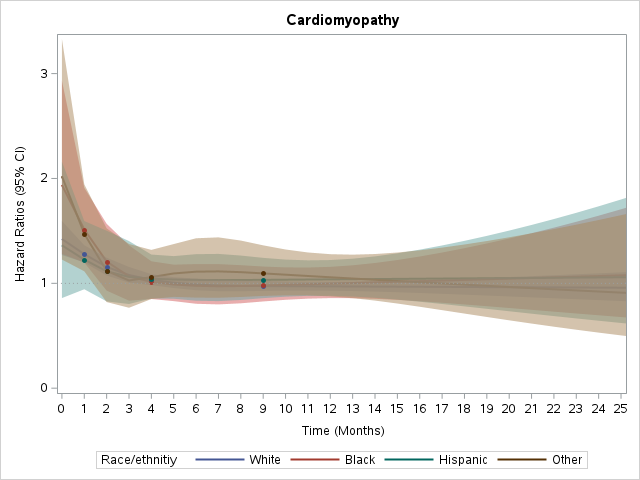


P=1.0 for interaction ^a^


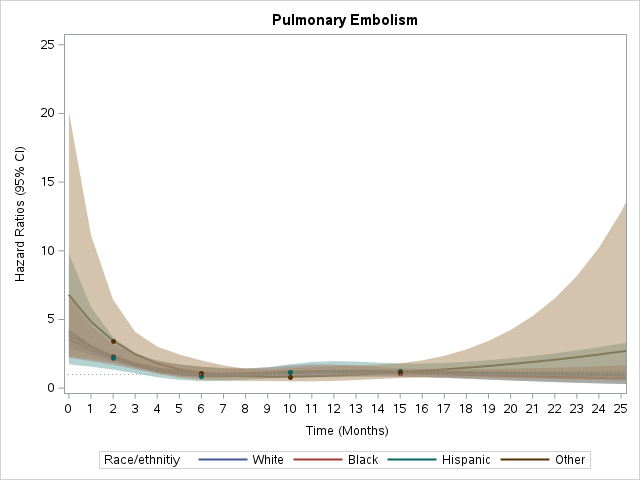


P=1.0 for interaction ^a^


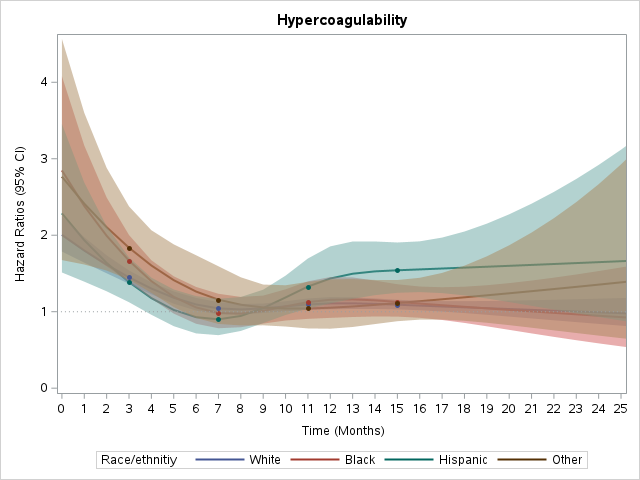


P=0.828 for interaction ^a^


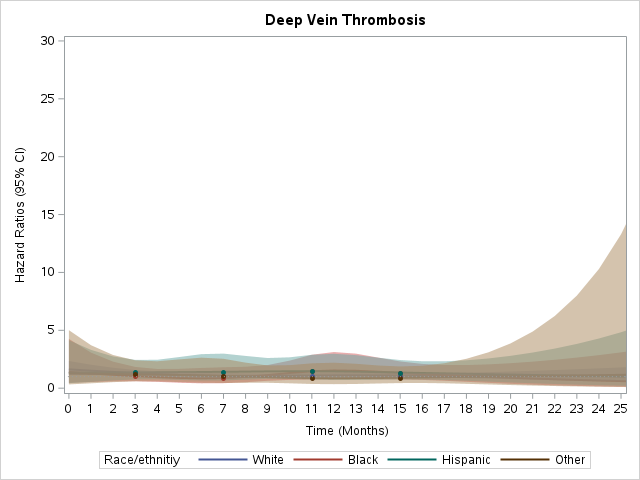


P=1.0 for interaction ^a^


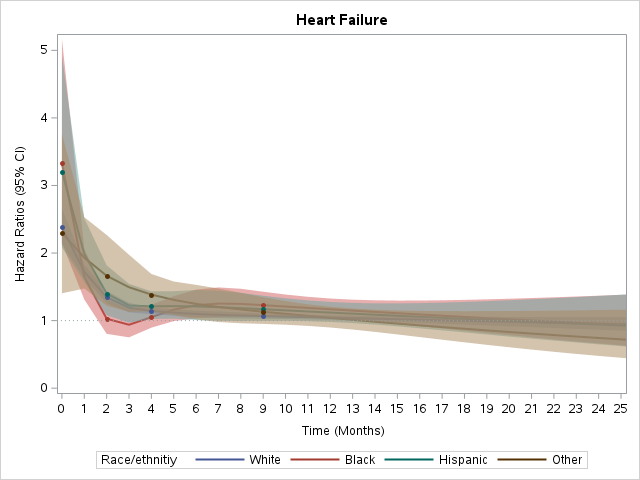


P=1.0 for interaction ^a^


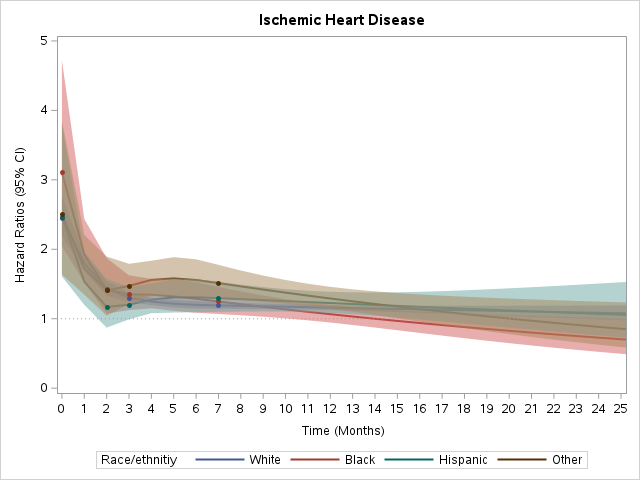


P=1.0 for interaction ^a^


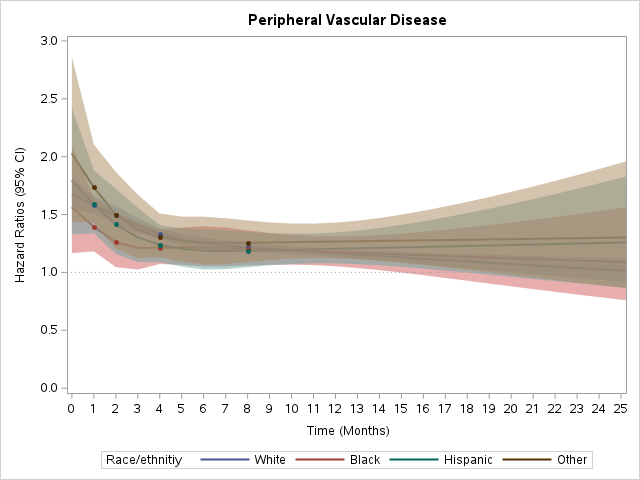


P=1.0 for interaction ^a^


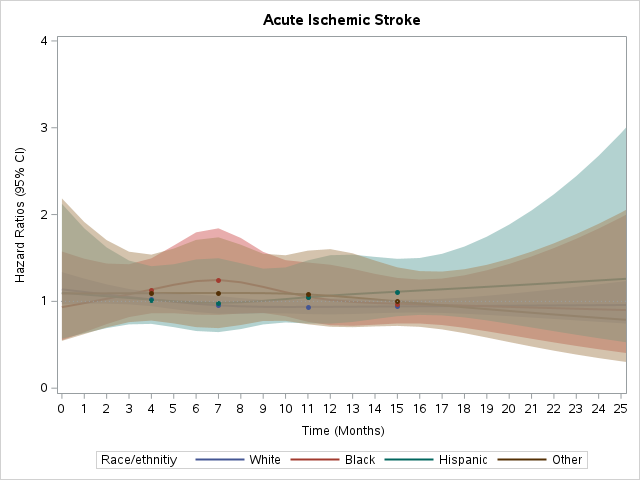


P=1.0 for interaction ^a^


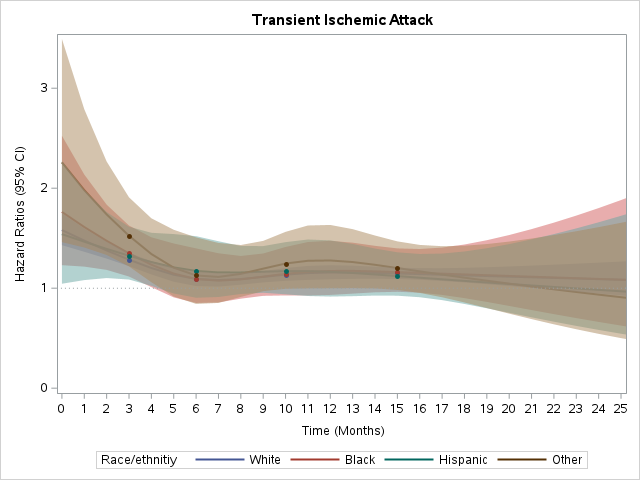


P=1.0 for interaction ^a^

^a^ P-values for interactions between COVID-19 and age group, sex and race/ethnicity on risk of mortality, hospitalization and incident CVD or stroke based on Bonferroni corrected p-values for multiple comparisons.
