# Supplementary material for: Long-term cardiovascular disease outcomes in non-hospitalized medicare beneficiaries diagnosed with COVID-19: Population-based matched cohort study
Source: PLoS One. 2024 May 14;19(5):e0302593. doi: 10.1371/journal.pone.0302593 (PMC11093379; doi:10.1371/journal.pone.0302593)

**S4 Fig. Cause-specific time-varying hazard ratios (95% CI) for risk of CVD and stroke associated with non-hospitalized COVID-19 including FFS beneficiaries with pre-existing CVD and stroke, Medicare 2020–2021 Matched Cohort**


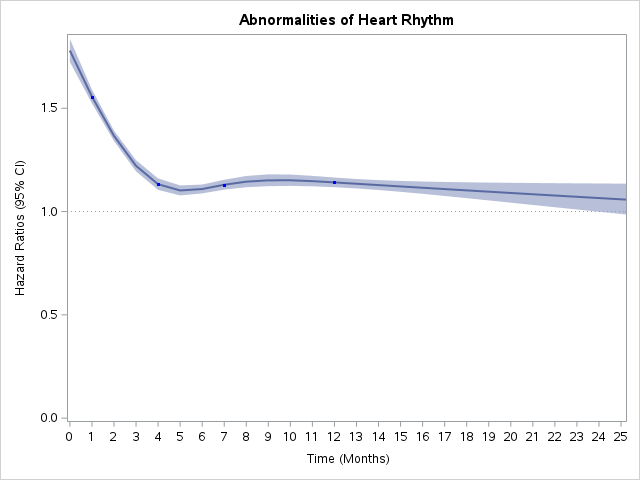


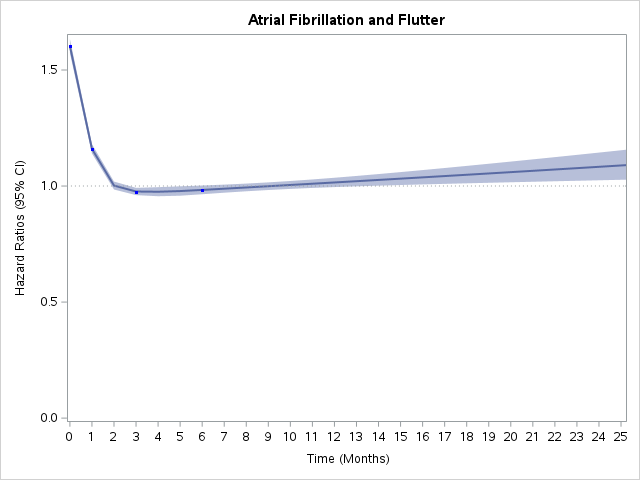


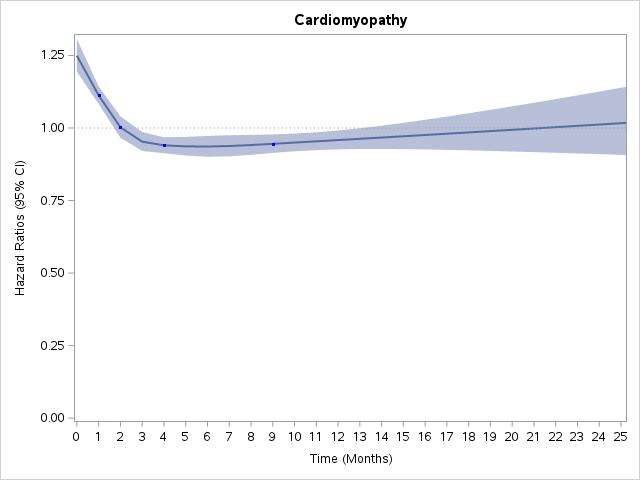


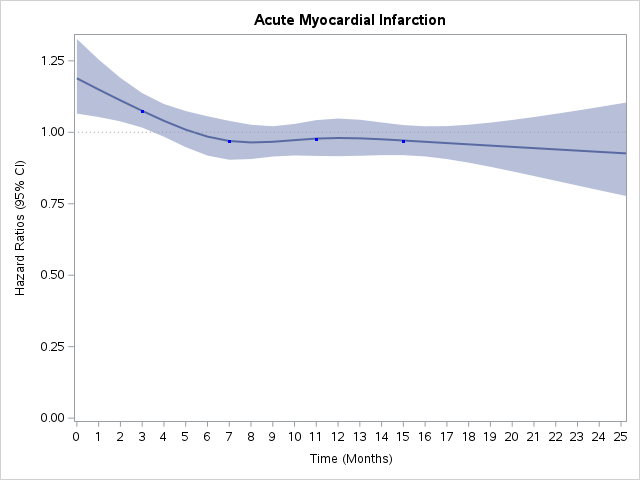


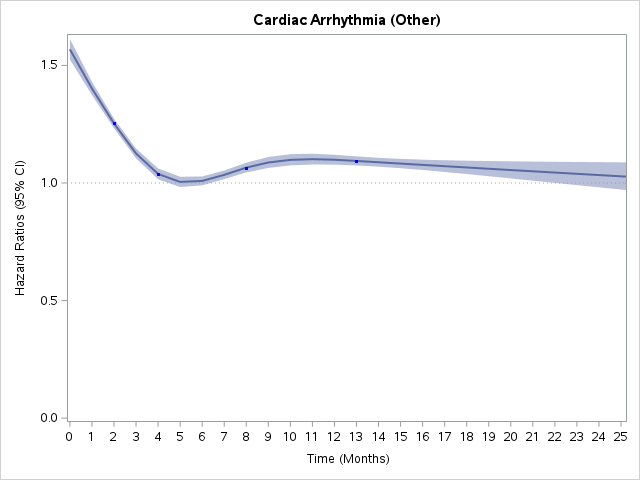


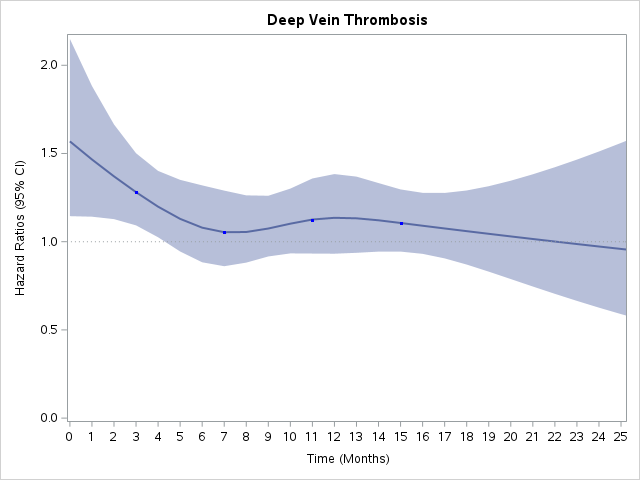


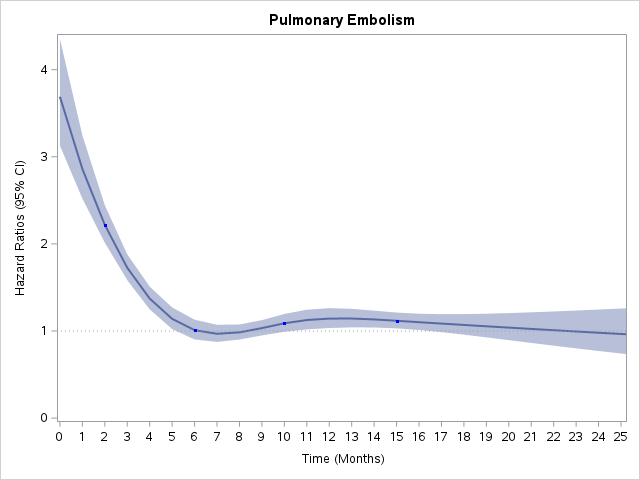


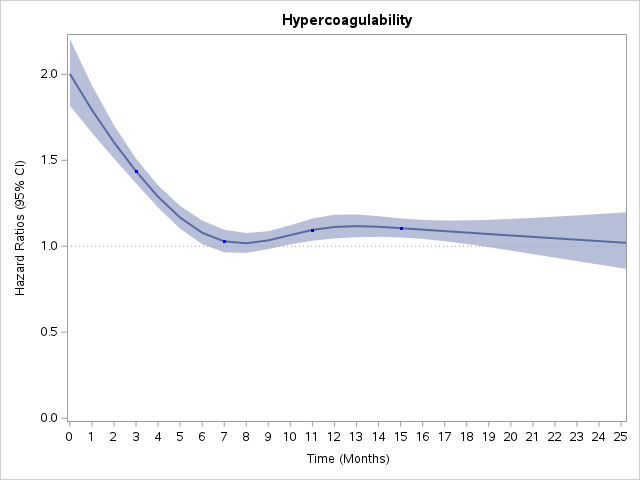


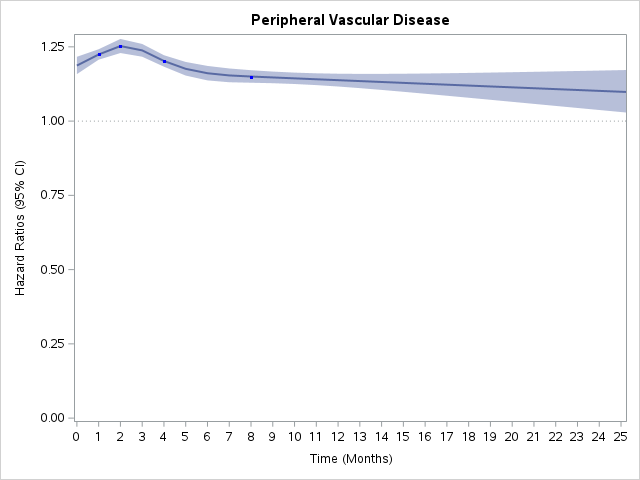


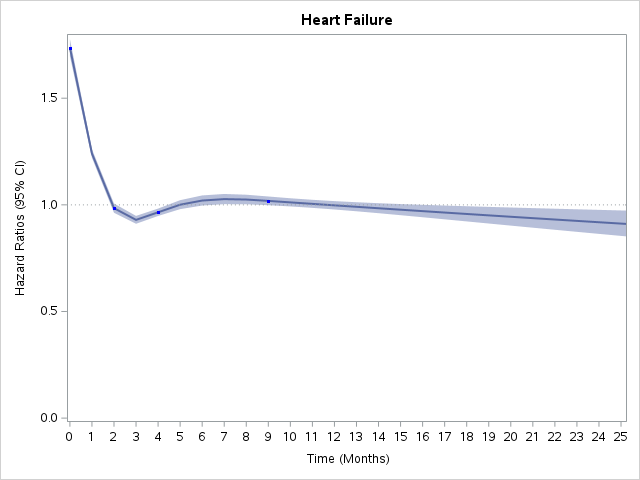


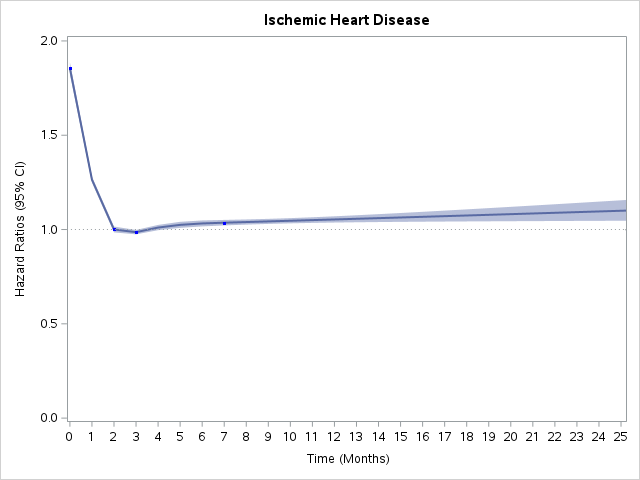


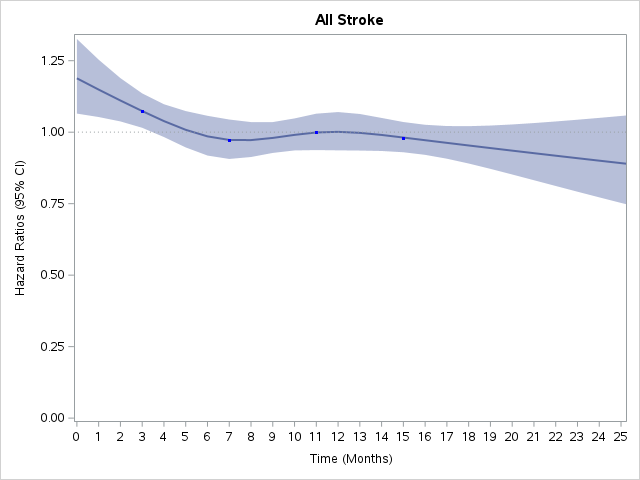


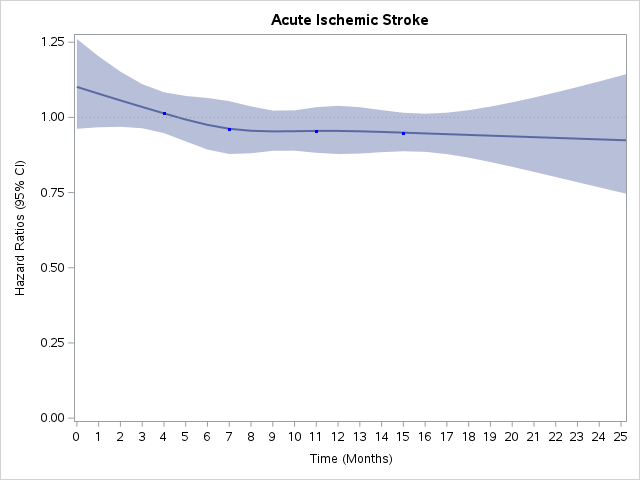


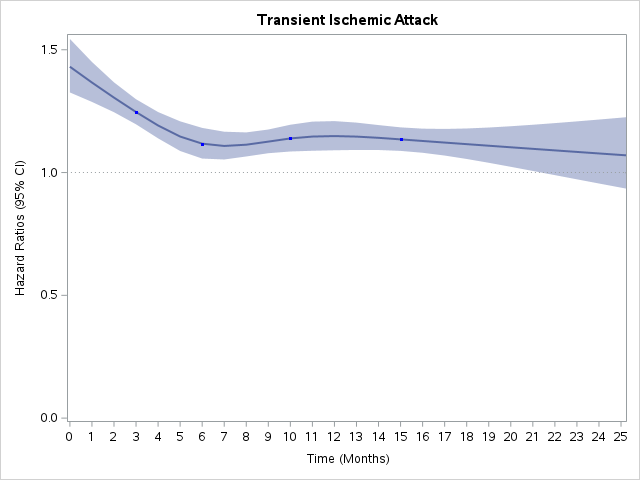


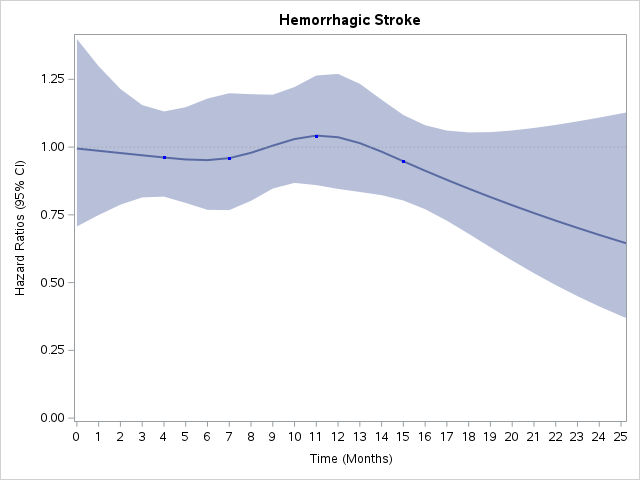

Supplement: S4 Fig — (DOCX) [file pone.0302593.s004.docx]
