# Supplementary material for: Long-term cardiovascular disease outcomes in non-hospitalized medicare beneficiaries diagnosed with COVID-19: Population-based matched cohort study
Source: PLoS One. 2024 May 14;19(5):e0302593. doi: 10.1371/journal.pone.0302593 (PMC11093379; doi:10.1371/journal.pone.0302593)
Supplement: S1 Table — (DOCX) [file pone.0302593.s005.docx]

**S1 Table. Covariates used in propensity score matching, Medicare 2020–2021 Matched Cohort**

| **Covariates** | **Demographic/Conditions** | **ICD-10 Diagnosis/Procedure and HCPCS Codes/Sources** |
| --- | --- | --- |
| Demographic factors | Age | -- |
|  | Sex | -- |
|  | Race/ethnicity | -- |
|  |  | -- |
| Socio-economic conditions | Metropolitan Statistical Area (urban/rural status) | -- |
|  | State of residence |  |
|  | Low Income Subsidy (LIS) Status | Yes/No |
|  | Dual eligibility for Medicare and Medicaid | Yes/No |
|  | Household Income by ZIP Code of Residence | Derived from American Community Surveys and matched to the baseline cohort years. United States Census Bureau: <https://factfinder.census.gov/faces/nav/jsf/pages/download_center.xhtml#none> |
|  | Social Vulnerability Index (2018) by US counties | CDC/ATSDR Social Vulnerability Index (SVI) by United States Census tracks, 2018: <https://www.atsdr.cdc.gov/placeandhealth/svi/data_documentation_download.html> |
| Healthcare utilization characteristics | Hospital outpatient visits |  |
|  | Home Health visits | -- |
|  | Hospital outpatient ER visits | -- |
|  | Inpatient ER visits | -- |
|  | Ambulatory Surgical Center Events | -- |
|  | Medicare Part B physician events | -- |
|  | Acute inpatient stays | -- |
|  | Other inpatient stays | -- |
|  | Service type |  |
|  | Flu Vaccine Indicator | HCPCS: 90470, 90653, 90655, 90656, 90657, 90658, 90659, 90660, 90661, 90662, 90663, 90672, 90674, 90682, 90685, 90686, 90687, 90688, 90694, 90724, 90756, G0008, G9141, G9142, Q2035 |
|  | Pneumococcal Vaccine Indicator | HCPCS: 90669, 90732, G0009 |
| Frailty characteristics | Home Oxygen | ICD-10: Z99.81; HCPCS: 99503, 99504, E0424, E0425, E0430, E0431, E0433, E0434, E0435, E0439, E0440, E0441, E0442, E0443, E0444, E0445, E0550, E0560, E1390, E1391, E1392, E1405, E1406, K0671 |
|  | Urinary Catheter | ICD-10: T8351XA, Z46.6, 0T9B70Z, 0T9B80Z, 0T2BX0Z, 3C1ZX8Z, 0TPDX0Z; HCPCS: 51702, 51703, A4311, A4312, A4313, A4314, A4315, A4316, A4338, A4340, A4344, A4346, A4355 |
|  | Walker Use | HCPCS: E0130, E0135, E0140, E0141, E0143, E0144, E0147, E0148, E0149, E0154, E0155, E0156, E0157, E0158, E0159, L1520 |
|  | Wheelchair Use | ICD-10: Z99.3, E88.43, Z46.89; HCPCS: 97542, E0192, E0950, E0951, E0952, E0953, E0954, E0955, E0956, E0957, E0958, E0959, E0960, E0961, E0962, E0963, E0964, E0965, E0966, E0967, E0968, E0969, E0971, E0972, E0973, E0974, E0977, E0978, E0981, E0982, E0983, E0984, E0985, E0986, E0990, E0992, E0995, E1002, E1003, E1004, E1005, E1006, E1007, E1008, E1009, E1010, E1011, E1012, E1013, E1014, E1015, E1016, E1017, E1018, E1019, E1020, E1021, E1025, E1026, E1027, E1028, E1029, E1030, E1050, E1060, E1070, E1083, E1084, E1085, E1086, E1087, E1088, E1089, E1090, E1093, E1100, E1130, E1140, E1150, E1160, E1161, E1170, E1171, E1172, E1180, E1190, E1195, E1200, E1210, E1211, E1212, E1213, E1220, E1221, E1222, E1223, E1224, E1225, E1226, E1227, E1228, E1240, E1250, E1260, E1270, E1280, E1285, E1290, E1295, E1296, E1297, E1298 |
| The Centers for Medicare and Medicaid (CMS) Chronic Condition Warehouse (CCW) Conditions |  | Centers for Medicare & Medicaid Services: <https://www2.ccwdata.org/web/guest/condition-categories> Accessed November 18, 2021. |
|  | Acquired Hypothyroidism |  |
|  | Acute Myocardial Infarction |  |
|  | Alcohol Use  Disorders |  |
|  | Alzheimer's Disease |  |
|  | Alzheimer's Disease and Related Disorders or Senile Dementia |  |
|  | Anemia |  |
|  | Anxiety Disorders |  |
|  | Asthma |  |
|  | Atrial Fibrillation |  |
|  | Cataract |  |
|  | Chronic Kidney Disease |  |
|  | Chronic Obstructive Pulmonary Disease and Bronchiectasis |  |
|  | Colorectal Cancer |  |
|  | Depressive disorders |  |
|  | Diabetes |  |
|  | Drug Use Disorders |  |
|  | Fibromyalgia and Chronic Pain and Fatigue |  |
|  | Glaucoma |  |
|  | Heart Failure |  |
|  | Hip/Pelvic Fracture |  |
|  | Human  Immunodeficiency  Virus and/or  Acquired  Immunodeficiency  Syndrome  (HIV/AIDS) |  |
|  | Hyperlipidemia |  |
|  | Hypertension |  |
|  | Intellectual Disabilities and  Related Conditions |  |
|  | Ischemic Heart Disease |  |
|  | Lung Cancer |  |
|  | Liver Disease,  Cirrhosis, and Other  Liver Conditions  (except Viral  Hepatitis) |  |
|  | Migraine and  Chronic Headache |  |
|  | Mobility  Impairments |  |
|  | Osteoporosis |  |
|  | Obesity |  |
|  | Peripheral Vascular Disease |  |
|  | Personality  Disorders |  |
|  | Rheumatoid Arthritis/  Osteoarthritis (RA/OA) |  |
|  | Stroke/Transient  Ischemic Attack |  |
|  | Tobacco Use |  |
|  | End-Stage Renal  Disease (ESRD) |  |
| CMS Hierarchical Condition Category (CMS-HCC) risk score |  | Centers for Medicare & Medicaid Services. Risk adjustment—2020 Model Software/ICD-10 Mappings: [https:// www.cms.gov/Medicare/Health-Plans/MedicareAdvtgSpecRateStats/Risk-Adjustors-Items/Risk2020](https://www.cms.gov/Medicare/Health-Plans/MedicareAdvtgSpecRateStats/Risk-Adjustors-Items/RiskModel2019.html) Accessed March 18, 2021. |
| Charlson Comorbidity Index |  | Quan H, Sundararajan V, Halfon P, et al. Coding algorithms for defining comorbidities in ICD-9-CM and ICD-10 administrative data. Med Care. 2005;43(11):1130-1139 |
