# Supplementary material for: Long-term cardiovascular disease outcomes in non-hospitalized medicare beneficiaries diagnosed with COVID-19: Population-based matched cohort study
Source: PLoS One. 2024 May 14;19(5):e0302593. doi: 10.1371/journal.pone.0302593 (PMC11093379; doi:10.1371/journal.pone.0302593)
Supplement: S2 Table — (DOCX) [file pone.0302593.s006.docx]

**S2 Table. ICD-10, CPT4 and HCPCS codes of chronic and other conditions based on CMS Chronic Condition Warehouse Condition Algorithms (February 2021) and published literatures**

| **Cardiovascular outcomes** | **ICD-10/CPT4/HCPCS Codes** | **References**^a^ | **Number/Type of Claims to Qualify**^b^ |
| --- | --- | --- | --- |
| Abnormalities of Heart Rhythm | R00.0 Tachycardia, unspecified  R00.1 Bradycardia, unspecified  R00.2 Palpitations  R00.8 Other abnormalities of heartbeat  R00.9 Unspecified abnormalities of heartbeat | Centers for Medicare & Medicaid Services:  <https://www.cms.gov/Medicare/Coding/ICD10/Downloads/ICD10ClinicalConceptsCardiology1.pdf>  Fung KW, Baye F, Kapusnik-Uner J, McDonald CJ. Using Medicare data to assess the proarrhythmic risk of non-cardiac treatment drugs that prolong the qt interval in older adults: An observational cohort study. Drugs Real World Outcomes. 2021;8:173-185 | At least 1 inpatient claim in Part A OR HOP or Carrier claims in Part B with the diagnosis code at ANY position |
| Acute Myocardial Infarction | I21.01, I21.02, I21.09, I21.11, I21.19, I21.21, I21.29, I21.3, I21.4, I21.9, I21.A1, I21.A9, I22.0, I22.1, I22.2, I22.8, I22.9  (ONLY first or second DX on the claim) | Centers for Medicare & Medicaid Services: <https://www2.ccwdata.org/web/guest/condition-categories-chronic>  Mentz RJ, Newby LK, Neely B, Lucas JE, Pokorney SD, Rao MP, Jackson LR, 2nd, Grau-Sepulveda MV, Smerek MM, Barth P, et al. Assessment of administrative data to identify acute myocardial infarction in electronic health records. J Am Coll Cardiol. 2016;67:2441-2442.  Kiyota Y, Schneeweiss S, Glynn RJ, Cannuscio CC, Avorn J, Solomon DH. Accuracy of medicare claims-based diagnosis of acute myocardial infarction: Estimating positive predictive value on the basis of review of hospital records. Am Heart J. 2004;148:99-104 | At least 1 inpatient claim in Part A and only primary or second position diagnosis on the inpatient claims |
| Atrial Fibrillation and Flutter | I48.0, I48.1, I48.11, I48.19, I48.2, I48.20, I48.21, I48.3, I48.4, I48.91  (ANY DX on the claims) | Centers for Medicare & Medicaid Services: <https://www2.ccwdata.org/web/guest/condition-categories-chronic>  Gage BF, Boechler M, Doggette AL, Fortune G, Flaker GC, Rich MW, Radford MJ. Adverse outcomes and predictors of underuse of antithrombotic therapy in Medicare beneficiaries with chronic atrial fibrillation. Stroke. 2000;31:822-827  Piccini JP, Hammill BG, Sinner MF, Jensen PN, Hernandez AF, Heckbert SR, Benjamin EJ, Curtis LH. Incidence and prevalence of atrial fibrillation and associated mortality among medicare beneficiaries, 1993-2007. Circ Cardiovasc Qual Outcomes. 2012;5:85-93. | At least 1 inpatient or SNF or HHA claims in Part A OR 2 HOP or Carrier claims in Part B with the diagnosis codes at ANY position (primary or secondary) on the claims |
| Cardiac Arrhythmia | I49.01 Ventricular fibrillation  I49.02 Ventricular flutter  I49.1 Atrial premature depolarization  I49.2 Junctional premature depolarization  I49.3 Ventricular premature depolarization  I49.40 Unspecified premature depolarization  I49.49 Other premature depolarization  I49.5 Sick sinus syndrome  I49.8 Other specified cardiac arrhythmias  I49.9 Cardiac arrhythmia, unspecified | Centers for Medicare & Medicaid Services:  <https://www.cms.gov/Medicare/Coding/ICD10/Downloads/ICD10ClinicalConceptsCardiology1.pdf>  Daugherty SE, Guo Y, Heath K, Dasmarinas MC, Jubilo KG, Samranvedhya J, Lipsitch M, Cohen K. Risk of clinical sequelae after the acute phase of sars-cov-2 infection: Retrospective cohort study. BMJ (Online). 2021;373:n1098  Al-Aly Z, Xie Y, Bowe B. High-dimensional characterization of post-acute sequelae of covid-19. Nature. 2021;594:259-264.  Fung KW, Baye F, Kapusnik-Uner J, McDonald CJ. Using Medicare data to assess the proarrhythmic risk of non-cardiac treatment drugs that prolong the qt interval in older adults: An observational cohort study. Drugs Real World Outcomes. 2021;8:173-185 | At least 1 inpatient claim in Part A OR HOP or Carrier claims in Part B with the diagnosis code at ANY position (primary or secondary diagnosis) |
| Cardiomyopathy | I25.5, I42.0, I42.1, I42.2, I42.3, I42.4, I42.5, I42.8, I42.9, I43, A36.81, B33.24 | Daugherty SE, Guo Y, Heath K, Dasmarinas MC, Jubilo KG, Samranvedhya J, Lipsitch M, Cohen K. Risk of clinical sequelae after the acute phase of sars-cov-2 infection: Retrospective cohort study. BMJ (Online). 2021;373:n1098  Chen J, Long JB, Hurria A, Owusu C, Steingart RM, Gross CP. Incidence of heart failure or cardiomyopathy after adjuvant trastuzumab therapy for breast cancer. J Am Coll Cardiol. 2012;60:2504-2512 | At least 1 inpatient claim in Part A or 2 HOP or Carrier claims in Part B at least 30 days apart with the diagnosis codes at ANY position |
| Deep Vein Thrombosis (DVT) | I80.1, I80.2, I80.3, I80.8, I80.9, I81, I82.0, I82.1, I82.2, I82.3, I82.8, I82.9 | Daugherty SE, Guo Y, Heath K, Dasmarinas MC, Jubilo KG, Samranvedhya J, Lipsitch M, Cohen K. Risk of clinical sequelae after the acute phase of sars-cov-2 infection: Retrospective cohort study. BMJ (Online). 2021;373:n1098  Abdul Sultan A, West J, Stephansson O, et al.  Defining venous thromboembolism and measuring its incidence using Swedish health registries: a nationwide pregnancy cohort study. BMJ Open 2015;5:e008864.  Alotaibi GS, Wu C, Senthilselvan A, McMurtry MS. The validity of ICD codes coupled with imaging procedure codes for identifying acute venous thromboembolism using administrative data. Vasc Med. 2015;20:364-368. | At least 1 inpatient claim in Part A with diagnosis codes at ANY position |
| Pulmonary Embolism (PE) | I26.0, I26.9 | Daugherty SE, Guo Y, Heath K, Dasmarinas MC, Jubilo KG, Samranvedhya J, Lipsitch M, Cohen K. Risk of clinical sequelae after the acute phase of sars-cov-2 infection: Retrospective cohort study. BMJ (Online). 2021;373:n1098  Abdul Sultan A, West J, Stephansson O, et al.  Defining venous thromboembolism and measuring its incidence using Swedish health registries: a nationwide pregnancy cohort study. BMJ Open 2015;5:e008864.  Alotaibi GS, Wu C, Senthilselvan A, McMurtry MS. The validity of ICD codes coupled with imaging procedure codes for identifying acute venous thromboembolism using administrative data. Vasc Med. 2015;20:364-368. | At least 1 inpatient claim in Part A with diagnosis codes at ANY position |
| Heart Failure | I09.81, I11.0, I13.0, I13.2, I50.1, I50.20, I50.21, I50.22, I50.23, I50.30, I50.31, I50.32, I50.33, I50.40, I50.41, I50.42, I50.43, I50.810, I50.811, I50.812, I50.813, I50.814, I50.82, I50.83, I50.84, I50.89, I50.9  (any DX on the claims) | Centers for Medicare & Medicaid Services: https://www2.ccwdata.org/web/guest/condition-categories-chronic  Chen J, Long JB, Hurria A, Owusu C, Steingart RM, Gross CP. Incidence of heart failure or cardiomyopathy after adjuvant trastuzumab therapy for breast cancer. J Am Coll Cardiol. 2012;60:2504-2512.  Khera R, Pandey A, Ayers CR, Agusala V, Pruitt SL, Halm EA, Drazner MH, Das SR, de Lemos JA, Berry JD. Contemporary epidemiology of heart failure in fee-for-service Medicare beneficiaries across healthcare settings. Circ Heart Fail. 2017;10:e004402. | Among FFS beneficiaries without historical diagnosis of heart failure, the incident heart failure was defined by either at least 1 inpatient or SNF or HHA claims in Part A with the diagnosis codes at ANY position OR 2 HOP or Carrier claims with diagnosis codes 30-day apart in Part B at ANY position.  To determine the date of heart failure onset, we used the  earlier of the following: (1) the date of the earliest inpatient  heart failure diagnosis or (2) the date of the earliest HOP or Carrier heart failure diagnosis. |
| Hypercoagulability | D68, I82 | Daugherty SE, Guo Y, Heath K, Dasmarinas MC, Jubilo KG, Samranvedhya J, Lipsitch M, Cohen K. Risk of clinical sequelae after the acute phase of sars-cov-2 infection: Retrospective cohort study. BMJ (Online). 2021;373:n1098. | At least 1 inpatient claim in Part A with the diagnosis codes at ANY position |
| Ischemic Heart Disease | I20.0, I20.1, I20.8, I20.9, I21.01, I21.02, I21.09, I21.11, I21.19, I21.21, I21.29, I21.3, I21.4, I21.A1, I21.A9, I22.0, I22.1, I22.2, I22.8, I22.9, I23.0, I23.1, I23.2, I23.3, I23.4, I23.5, I23.6, I23.7, I23.8, I24.0, I24.1, I24.8, I24.9, I25.10, I25.110, I25.111, I25.118, I25.119, I25.2, I25.3, I25.41, I25.42, I25.5, I25.6, I25.700, I25.701, I25.708, I25.709, I25.710, I25.711, I25.718, I25.719, I25.720, I25.721, I25.728, I25.729, I25.730, I25.731, I25.738, I25.739, I25.750, I25.751, I25.758, I25.759, I25.760, I25.761, I25.768, I25.769, I25.790, I25.791, I25.798, I25.799, I25.810, I25.811, I25.812, I25.82, I25.83, I25.84, I25.89, I25.9  (any DX on the claim) | Centers for Medicare & Medicaid Services: <https://www2.ccwdata.org/web/guest/condition-categories-chronic>  Hlatky MA, Ray RM, Burwen DR, Margolis KL, Johnson KC, Kucharska-Newton A, Manson JE, Robinson JG, Safford MM, Allison M, et al. Use of medicare data to identify coronary heart disease outcomes in the women's health initiative. Circ Cardiovasc Qual Outcomes. 2014;7:157-162. | At least 1 inpatient or SNF or HHA claims in Part A  OR 2 HOP or Carrier claims in Part B with the diagnosis codes at ANY position on the claims. |
| Peripheral Vascular Disease (PVD) | E08.51, E08.52, E09.51, E09.52, E10.51, E10.52, E11.51, E11.52, E13.51, E13.52, I70.0, I70.1, I70.201, I70.202, I70.203, I70.208, I70.209, I70.211, I70.212, I70.213, I70.218, I70.219, I70.221, I70.222, I70.223, I70.228, I70.229, I70.231, I70.232, I70.233, I70.234, I70.235, I70.238, I70.239, I70.241, I70.242, I70.243, I70.244, I70.245, I70.248, I70.249, I70.25, I70.291, I70.292, I70.293, I70.298, I70.299, I70.92, I73.81, I73.89, I73.9, I79.1, I79.8 (any DX on the claim) | Centers for Medicare & Medicaid Services: <https://www2.ccwdata.org/web/guest/condition-categories-other>  Kalbaugh CA, Kucharska-Newton A, Wruck L, Lund JL, Selvin E, Matsushita K, Bengtson LGS, Heiss G, Loehr L. Peripheral artery disease prevalence and incidence estimated from both outpatient and inpatient settings among Medicare fee-for-service beneficiaries in the atherosclerosis risk in communities (ARIC) study. J Am Heart Assoc. 2017;6(5). | At least 1 inpatient claim in Part A OR 2 HOP or Carrier claims in Part B with the diagnosis codes at ANY position |
| **Cerebrovascular outcomes** |  |  |  |
| All stroke | I60, I61, I62, I63, I64, I65, I66, I67, I68, I69 | Daugherty SE, Guo Y, Heath K, Dasmarinas MC, Jubilo KG, Samranvedhya J, Lipsitch M, Cohen K. Risk of clinical sequelae after the acute phase of sars-cov-2 infection: Retrospective cohort study. BMJ (Online). 2021;373:n1098  Al-Aly Z, Xie Y, Bowe B. High-dimensional characterization of post-acute sequelae of covid-19. Nature. 2021;594:259-264  Yang Q, Chang A, Tong X, Merritt R. Herpes zoster vaccine live and risk of stroke among Medicare beneficiaries: A population-based cohort study. Stroke. 2021;52:1712-1721 | At least 1 inpatient claim in Part A with the primary diagnosis on the claims |
| Acute Ischemic Stroke | I63 | Daugherty SE, Guo Y, Heath K, Dasmarinas MC, Jubilo KG, Samranvedhya J, Lipsitch M, Cohen K. Risk of clinical sequelae after the acute phase of sars-cov-2 infection: Retrospective cohort study. BMJ (Online). 2021;373:n1098  Al-Aly Z, Xie Y, Bowe B. High-dimensional characterization of post-acute sequelae of covid-19. Nature. 2021;594:259-264  Yang Q, Chang A, Tong X, Merritt R. Herpes zoster vaccine live and risk of stroke among Medicare beneficiaries: A population-based cohort study. Stroke. 2021;52:1712-1721. | At least 1 inpatient claim in Part A with the primary diagnosis on the claims |
| Hemorrhagic stroke | I60, I61 | Daugherty SE, Guo Y, Heath K, Dasmarinas MC, Jubilo KG, Samranvedhya J, Lipsitch M, Cohen K. Risk of clinical sequelae after the acute phase of sars-cov-2 infection: Retrospective cohort study. BMJ (Online). 2021;373:n1098  Al-Aly Z, Xie Y, Bowe B. High-dimensional characterization of post-acute sequelae of covid-19. Nature. 2021;594:259-264  Yang Q, Chang A, Tong X, Merritt R. Herpes zoster vaccine live and risk of stroke among Medicare beneficiaries: A population-based cohort study. Stroke. 2021;52:1712-1721. | At least 1 inpatient claim in Part A with the primary diagnosis on the claims |
| Transient Ischemic Attack (TIA) | G45.0, G45.1, G45.2, G45.8, G45.9 | Clinical Classifications Software Refined (CCSR) for ICD-10-CM Diagnoses: <https://www.hcup-us.ahrq.gov/toolssoftware/ccsr/dxccsr.jsp>  Wilcock AD, Zachrison KS, Schwamm LH, Uscher-Pines L, Zubizarreta JR, Mehrotra A. Trends among rural and urban medicare beneficiaries in care delivery and outcomes for acute stroke and transient ischemic attacks, 2008-2017. JAMA Neurol. 2020;77:863-871. | At least 1 inpatient claim in Part A OR HOP or Carrier claims in Part B with primary diagnosis codes on the claims |

^a^ For conditions that were included in Centers for Medicare & Medicaid Services (CMS) Chronic Condition Warehouse (CCW), such as acute myocardial infarction, ischemic heart diseases, heart failure, we used the ICD-10/CPT4/HCPCS codes provided by CMS for CCW Condition Algorithms. For conditions that were not included in CCW Condition Algorithms, we used the ICD-10 codes provided in the published literatures.

^b^ The notes included the detailed information on type of Medicare claims (Part A or Part B), number of events and diagnosis positions to identify the cardiovascular diseases/conditions. SNF refers to skilled nursing facility; HHA refers to home health agency; HOP refers to hospital outpatient. Carrier claims refer to claim types 71 and 72 (not durable medical equipment [DME] claim types 81 or 82) and excludes any claims for which line-item Berenson-Eggers Type of Service (BETOS) code variable equals D1A, D1B, D1C, D1D, D1E, D1F, D1G (which is DME), or O1A (which is ambulance services). The intent of the algorithm is to exclude claims where the services do not require a licensed health care professional. When two claims are required, they must occur at least one day apart.
