# Supplementary material for: Long-term cardiovascular disease outcomes in non-hospitalized medicare beneficiaries diagnosed with COVID-19: Population-based matched cohort study
Source: PLoS One. 2024 May 14;19(5):e0302593. doi: 10.1371/journal.pone.0302593 (PMC11093379; doi:10.1371/journal.pone.0302593)
Supplement: S3 Table — (DOCX) [file pone.0302593.s007.docx]

**S3 Table. Characteristics of original cohort Medicare FFS beneficiaries aged 66 years or older compared to non-hospitalized COVID-19 beneficiaries and matched controls, Medicare 2020-2021 Matched Cohort**

| **Characteristics** | **Number of Medicare FFS beneficiaries without COVID-19** | **Mean / %  (95% CI)** | **Number of Medicare FFS beneficiaries with Non-hospitalized COVID-19** | **Non-hospitalized COVID-19 mean/% (95% CI)** | **Number of matched Medicare FFS beneficiaries without COVID-19** | **Without COVID-19 mean/%  (95% CI)** | **Differences between Medicare FFS beneficiaries without COVID-19 and with Non-hospitalized COVID-19 ^a^** | | **Standardized differences between non-hospitalized COVID-19 and matched FFS beneficiaries ^b^** |
| --- | --- | --- | --- | --- | --- | --- | --- | --- | --- |
| **All** | 22,003,189 |  | 944,371 |  | 944,371 |  |  | |  |
| Age, mean |  | 74.82  (74.82-74.83) |  | 75.28  (75.26-75.29) |  | 75.28  (75.26-75.29) | 0.0602 | | 0.0000 |
| Age group (%) |  |  |  |  |  |  |  | |  |
| 66–74 years | 12,477,890 | 56.71  (56.69-56.73) | 524,925 | 55.58  (55.48-55.68) | 524,925 | 55.58  (55.48-55.68) | -0.0301 | | 0.000 |
| 75–84 years | 6,821,581 | 31.00  (30.98-31.02) | 296,336 | 31.38  (31.29-31.47) | 296,336 | 31.38  (31.29-31.47) | 0.0081 | | 0.000 |
| ≥85 years | 2,703,718 | 12.29  (12.27-12.30) | 123,110 | 13.04  (12.97-13.10) | 123,110 | 13.04  (12.97-13.10) | 0.0334 | | 0.000 |
| **Sex (%)** |  |  |  |  |  |  |  | |  |
| Men | 9,734,886 | 44.24  (44.22-44.26) | 396,517 | 41.99  (41.89-42.09) | 396,517 | 41.99  (41.89-42.09) |  | |  |
| Women | 12,268,303 | 55.76  (55.74-55.78) | 547,854 | 58.01  (57.91-58.11) | 547,854 | 58.01  (57.91-58.11) | 0.0471 | | 0.000 |
| **Race/ethnicity** |  |  |  |  |  |  |  | |  |
| Non-Hispanic White | 18,108,387 | 82.30  (82.28-82.31) | 779,549 | 82.55  (82.47-82.62) | 779,549 | 82.55  (82.47-82.62) | 0.0048 | | 0.000 |
| Non-Hispanic Black | 1,454,090 | 6.61  (6.60- 6.62) | 56,030 | 5.93  (5.89- 5.98) | 56,030 | 5.93 (  5.89- 5.98) | -0.0256 | | 0.000 |
| Hispanic | 1,045,824 | 4.75  (4.74- 4.76) | 58,264 | 6.17  (6.12- 6.22) | 58,264 | 6.17  (6.12- 6.22) | 0.0630 | | 0.000 |
| Other | 1,394,888 | 6.34  (6.33- 6.35) | 50,528 | 5.35  (5.31- 5.40) | 50,528 | 5.35  (5.31- 5.40) | -0.0424 | | 0.000 |
| **Low-income subsidy (%)** |  |  |  |  |  |  |  | |  |
| Yes | 2,432,511 | 11.06  (11.04-11.07) | 156,178 | 16.54  (16.46-16.61) | 151,828 | 16.08  (16.00-16.15) | 0.1727 | | 0.0125 |
| No | 19,570,678 | 88.94  (88.93-88.96) | 788,193 | 83.46  (83.39-83.54) | 792,543 | 83.92  (83.85-84.00) |  | |  |
| **Household Income, Median (IQR)** |  | 77,111  (62,080-101,398) |  | 75,566  (60,954-100,909) |  | 76,288  (61,370-100,973) | -0.0219 | | -0.0063 |
| **Social Vulnerability Index, Median (IQR)** |  | 0.51  (0.28-0.69) |  | 0.54  (0.29-0.74) |  | 0.53  (0.31-0.73) | 0.0977 | | 0.0008 |
| **Ischemic Heart Disease (%)** |  |  |  |  |  |  |  | |  |
| Yes | 8,606,888 | 39.12  (39.10-39.14) | 455,368 | 48.22  (48.12-48.32) | 449,400 | 47.59  (47.49-47.69) | 0.1891 | | 0.0127 |
| No | 13,396,301 | 60.88  (60.86-60.90) | 489,003 | 51.7  (51.68-51.88) | 494,971 | 52.41  (52.31-52.51) |  | |  |
| **Acute Myocardial Infarction (%)** |  |  |  |  |  |  |  | |  |
| Yes | 909,328 | 4.13  (4.12- 4.14) | 43,297 | 4.58  (4.54- 4.63) | 40,799 | 4.32  (4.28- 4.36) | 0.0248 | | 0.0128 |
| No | 21,093,861 | 95.87  (95.86-95.88) | 901,074 | 95.42  (95.37-95.46) | 903,572 | 95.68  (95.64-95.72) |  | |  |
| **Congestive heart failure (%)** |  |  |  |  |  |  |  | |  |
| Yes | 4,278,134 | 19.44  (19.43-19.46) | 237,298 | 25.13  (25.04-25.22) | 226,453 | 23.98  (23.89-24.07) | 0.1447 | | 0.0267 |
| No | 17,725,055 | 80.56  (80.54-80.57) | 707,073 | 74.87  (74.78-74.96) | 717,918 | 76.02  (75.93-76.11) |  | |  |
| **Peripheral Vascular Disease (%)** |  |  |  |  |  |  |  | |  |
| Yes | 4,026,604 | 18.30  (18.28-18.32) | 242,314 | 25.66  (25.57-25.75) | 232,911 | 24.66  (24.58-24.75) | 0.1876 | | 0.0229 |
| No | 17,976,585 | 81.70  (81.68-81.72) | 702,057 | 74.34  (74.25-74.43) | 711,460 | 75.34  (75.25-75.42) |  | |  |
| **Atrial fibrillation (%)** |  |  |  |  |  |  |  | |  |
| Yes | 2,964,137 | 13.47  (13.46-13.49) | 149,813 | 15.86  (15.79-15.94) | 144,560 | 15.31  (15.23-15.38) | 0.0715 | | 0.0153 |
| No | 19,039,052 | 86.53  (86.51-86.54) | 794,558 | 84.14  (84.06-84.21) | 799,811 | 84.69  (84.62-84.77) |  | |  |
| **Hypertension (%)** |  |  |  |  |  |  |  | |  |
| Yes | 16,269,132 | 73.94  (73.92-73.96) | 769,036 | 81.43  (81.36-81.51) | 773,812 | 81.94  (81.86-82.02) | 0.1844 | | -0.0131 |
| No | 5,734,057 | 26.06 (26.04-26.08) | 175,335 | 18.57 (18.49-18.64) | 170,559 | 18.06 (17.98-18.14) |  | |  |
| **Hyperlipidemia (%)** |  |  |  |  |  |  |  | |  |
| Yes | 16,464,962 | 74.83  (74.81-74.85) | 780,417 | 82.64  (82.56-82.72) | 786,715 | 83.31  (83.23-83.38) | 0.1932 | | -0.0177 |
| No | 5,538,227 | 25.17  (25.15-25.19) | 163,954 | 17.36  (17.28-17.44) | 157,656 | 16.69  (16.62-16.77) |  | |  |
| **Stroke/TIA (%)** |  |  |  |  |  |  |  | |  |
| Yes | 2,510,846 | 11.41  (11.40-11.42) | 138,937 | 14.71  (14.64-14.78) | 133,209 | 14.11  (14.04-14.18) | 0.1047 | | 0.0173 |
| No | 19,492,343 | 88.59  (88.58-88.60) | 805,434 | 85.29  (85.22-85.36) | 811,162 | 85.89  (85.82-85.96) |  | |  |
| **Diabetes (%)** |  |  |  |  |  |  |  | |  |
| Yes | 7,053,559 | 32.06  (32.04-32.08) | 376,471 | 39.86  (39.77-39.96) | 373,032 | 39.50  (39.40-39.60) | 0.1669 | | 0.0074 |
| No | 14,949,630 | 67.94  (67.92-67.96) | 567,900 | 60.14 (60.04-60.23) | 571,339 | 60.50  (60.40-60.60) |  | |  |
| **COPD (%)** |  |  |  |  |  |  |  | |  |
| Yes | 4,452,914 | 20.24  (20.22-20.25) | 235,648 | 24.95  (24.87-25.04) | 225,656 | 23.89  (23.81-23.98) | 0.1178 | | 0.0239 |
| No | 17,550,275 | 79.76  (79.75-79.78) | 708,723 | 75.05  (74.96-75.13) | 718,715 | 76.11  (76.02-76.19) |  | |  |
| **Alzheimer (%)** |  |  |  |  |  |  |  | |  |
| Yes | 804,902 | 3.66  (3.65- 3.67) | 70,488 | 7.46  (7.41- 7.52) | 60,717 | 6.43  (6.38- 6.48) | 0.1778 | | 0.0407 |
| No | 21,198,287 | 96.34  (96.33-96.35) | 873,883 | 92.54  (92.48-92.59) | 883,654 | 93.57  (93.52-93.62) |  | |  |
| **Obesity (%)** |  |  |  |  |  |  |  | |  |
| Yes | 6,062,487 | 27.55  (27.53-27.57) | 325,046 | 34.42  (34.32-34.52) | 325,857 | 34.51  (34.41-34.60) | 0.1489 | | -0.0018 |
| No | 15,940,702 | 72.45  (72.43-72.47) | 619,325 | 65.58  (65.48-65.68) | 618,514 | 65.49  (65.40-65.59) |  | |  |
| **Tobacco use (%)** |  |  |  |  |  |  |  | |  |
| Yes | 2,510,300 | 11.41  (11.40-11.42) | 96,861 | 10.26  (10.20-10.32) | 92,763 | 9.82  (9.76- 9.88) | -0.0351 | | 0.0144 |
| No | 19,492,889 | 88.59  (88.58-88.60) | 847,510 | 89.74  (89.68-89.80) | 851,608 | 90.18  (90.12-90.24) |  | |  |
| **Charlson-Comorbidity Index (%)** |  |  |  |  |  |  | |  |  |
| 0 | 18,638,875 | 84.71  (84.69-84.72) | 748,533 | 81.80  (81.72-81.88) | 772,815 | 84.45  (84.38-84.52) | -0.0784 | | -0.0709 |
| 1 | 897,889 | 4.08  (4.07- 4.09) | 775,040 | 82.07  (81.99-82.15) | 799,939 | 84.71  (84.63-84.78) | 0.0432 | | 0.0202 |
| 2 | 725,510 | 3.30  (3.29- 3.30) | 46,675 | 4.94  (4.90- 4.99) | 42,624 | 4.51  (4.47- 4.56) | 0.0344 | | 0.0227 |
| 3 | 502,429 | 2.28  (2.28- 2.29) | 36,765 | 3.89  (3.85- 3.93) | 32,721 | 3.46  (3.43- 3.50) | 0.0327 | | 0.0248 |
| 4 | 357,379 | 1.62  (1.62- 1.63) | 26,008 | 2.75  (2.72- 2.79) | 22,305 | 2.36  (2.33- 2.39) | 0.0248 | | 0.0235 |
| 5+ | 881,107 | 4.00  (4.00- 4.01) | 18,094 | 1.92  (1.89- 1.94) | 15,180 | 1.61  (1.58- 1.63) | 0.0268 | | 0.0558 |

Abbreviations: CI, confidence interval; COPD, chronic obstructive pulmonary disease; FFS, fee-for-service; IQR, interquartile range; TIA, transient ischemic stroke.

^a^ Differences in means or proportions between Medicare FFS beneficiaries without COVID-19 and non-hospitalized COVID-19 Medicare FFS beneficiaries.

^b^ Standardized differences were the differences in means or proportions between non-hospitalized COVID-19 and matched Medicare FFS beneficiaries divided by standard errors where differences <0.10 were considered negligible.
