# Supplementary material for: Long-term cardiovascular disease outcomes in non-hospitalized medicare beneficiaries diagnosed with COVID-19: Population-based matched cohort study
Source: PLoS One. 2024 May 14;19(5):e0302593. doi: 10.1371/journal.pone.0302593 (PMC11093379; doi:10.1371/journal.pone.0302593)
Supplement: S4 Table — (DOCX) [file pone.0302593.s008.docx]

**S4 Table.** **Incidence rates of any diagnosis and adjusted hazard ratio (95% CI) for risk of CVD and stroke associated with non-hospitalized COVID-19 by follow-up time (including FFS beneficiaries with pre-existing CVD and stroke), Medicare 2020–2021 Matched Cohort**

|  | **0-3 month** | | | **4-6 month** | | | **≥7 month** | | |
| --- | --- | --- | --- | --- | --- | --- | --- | --- | --- |
|  | **COVID-19 status** | | | **COVID-19 status** | | | **COVID-19 status** | | |
| **Conditions**^a^ | **Yes** | **No** | **HR (95% CI)** ^b^ | **Yes** | **No** | **HR (95% CI)** ^b^ | **Yes** | **No** | **HR (95% CI)** ^b^ |
| **Cardiovascular outcomes** |  |  |  |  |  |  |  |  |  |
| **Abnormality of heart rhythm** |  |  |  |  |  |  |  |  |  |
| Events (person-years) ^c^ | 44718 (228319) | 30019 (231243) |  | 22951 (219009) | 19787 (223214) |  | 45918 (837723) | 41265 (859778) |  |
| Incidence rate of any diagnosis per 1,000 person-years (95% CI) | 195.9  (194.1-197.7) | 129.8  (128.4-131.3) | 1.50  (1.47 - 1.53) | 104.8  (103.4-106.2) | 88.6  (87.4 - 89.9) | 1.18  (1.15 - 1.21) | 54.8  (54.3 - 55.3) | 48.0  (47.5 - 48.5) | 1.14  (1.12 - 1.16) |
| **AMI** |  |  |  |  |  |  |  |  |  |
| Events (person-years) ^c^ | 2424 (234690) | 2054 (234987) |  | 2205 (231172) | 2079 (231619) |  | 8119 (924783) | 7980 (926034) |  |
| Incidence rate of any diagnosis per 1,000 person-years (95% CI) | 10.3  (9.9 - 10.7) | 8.7  (8.4 - 9.1) | 1.14  (1.05 - 1.23) | 9.5  (9.1 - 9.9) | 9.0  (8.6 - 9.4) | 1.02  (0.94 - 1.11) | 8.8  (8.6 - 9.0) | 8.6  (8.4 - 8.8) | 0.98  (0.94 - 1.03) |
| **AFIB and Flutter** |  |  |  |  |  |  |  |  |  |
| Events (person-years) ^c^ | 113373 (214844) | 94310 (220100) |  | 23326 (202591) | 26537 (207071) |  | 32303 (786817) | 33217 (801038) |  |
| Incidence rate of any diagnosis per 1,000 person-years (95% CI) | 527.7  (524.6-530.8) | 428.5  (425.8-431.2) | 1.31  (1.30 - 1.33) | 115.1  (113.7-116.6) | 128.2  (126.6-129.7) | 0.96  (0.93 - 0.98) | 41.1  (40.6 - 41.5) | 41.5  (41.0 - 41.9) | 1.02  (1.00 - 1.05) |
| **Cardiac Arrhythmia** |  |  |  |  |  |  |  |  |  |
| Events (person-years) ^c^ | 48960 (227831) | 36677 (230402) |  | 25485 (217918) | 23753 (221422) |  | 64675 (826561) | 60694 (843217) |  |
| Incidence rate of any diagnosis per 1,000 person-years (95% CI) | 214.9  (213.0-216.8) | 159.2  (157.6-160.8) | 1.34  (1.31 - 1.36) | 116.9  (115.5-118.4) | 107.3  (105.9-108.6) | 1.08  (1.06 - 1.11) | 78.2  (77.6 - 78.9) | 72.0  (71.4 - 72.6) | 1.08  (1.07 - 1.10) |
| **Cardiomyopathy** |  |  |  |  |  |  |  |  |  |
| Events (person-years) ^c^ | 22879 (231502) | 20670 (232230) |  | 8374 (225573) | 8601 (226416) |  | 11909 (895565) | 11996 (898026) |  |
| Incidence rate of any diagnosis per 1,000 person-years (95% CI) | 98.8  (97.6 -100.1) | 89.0  (87.8 - 90.2) | 1.08  (1.05 - 1.11) | 37.1  (36.3 - 37.9) | 38.0  (37.2 - 38.8) | 0.95  (0.91 - 0.99) | 13.3  (13.1 - 13.5) | 13.4  (13.1 - 13.6) | 0.97  (0.93 - 1.00) |
| **DVT** |  |  |  |  |  |  |  |  |  |
| Events (person-years) ^c^ | 343 (234931) | 233 (235178) |  | 305 (231755) | 272 (232134) |  | 1035 (930997) | 930  (931811) |  |
| Incidence rate of any diagnosis per 1,000 person-years (95% CI) | 1.5  (1.3 - 1.6) | 1.0  (0.9 - 1.1) | 1.42  (1.12 - 1.80) | 1.3  (1.2 - 1.5) | 1.2  (1.0 - 1.3) | 1.08  (0.86 - 1.36) | 1.1  (1.0 - 1.2) | 1.0  (0.9 - 1.1) | 1.07  (0.94 - 1.21) |
| **PE** |  |  |  |  |  |  |  |  |  |
| Events (person-years) ^c^ | 1985 (234690) | 755 (235130) |  | 978 (231358) | 832 (231990) |  | 3499 (928178) | 3190 (930128) |  |
| Incidence rate of any diagnosis per 1,000 person-years (95% CI) | 8.5  (8.1 - 8.8) | 3.2  (3.0 - 3.4) | 2.55  (2.26-2.87) | 4.2  (4.0 - 4.5) | 3.6  (3.4 - 3.8) | 1.14  (1.00 - 1.30) | 3.8  (3.6 - 3.9) | 3.4  (3.3 - 3.6) | 1.07  (1.00 - 1.14) |
| **HF** |  |  |  |  |  |  |  |  |  |
| Events (person-years) ^c^ | 78569 (221405) | 59936 (226068) |  | 20990 (211420) | 21654 (216181) |  | 34614 (822055) | 35185 (840370) |  |
| Incidence rate of any diagnosis per 1,000 person-years (95% CI) | 354.9  (352.4-357.4) | 265.1  (263.0-267.3) | 1.32  (1.30 - 1.34) | 99.3  (97.9 -100.6) | 100.2  (98.8 -101.5) | 0.98  (0.96 - 1.01) | 42.1  (41.7 - 42.6) | 41.9  (41.4 - 42.3) | 0.99  (0.97 - 1.01) |
| **Hypercoagulability** |  |  |  |  |  |  |  |  |  |
| Events (person-years) ^c^ | 3980 (234494) | 2157 (234986) |  | 2812 (230876) | 2472 (231613) |  | 10271 (922904) | 8948 (925999) |  |
| Incidence rate of any diagnosis per 1,000 person-years (95% CI) | 17.0  (16.5 - 17.5) | 9.2  (8.8 - 9.6) | 1.76  (1.63 - 1.89) | 12.2  (11.7 - 12.6) | 10.7  (10.3 - 11.1) | 1.09  (1.01 - 1.17) | 11.1  (10.9 - 11.3) | 9.7  (9.5 - 9.9) | 1.09  (1.05 - 1.14) |
| **IHD** |  |  |  |  |  |  |  |  |  |
| Events (person-years) ^c^ | 163934 (207368) | 129056 (216238) |  | 46771 (187824) | 50795 (196154) |  | 57215 (692514) | 58014 (722890) |  |
| Incidence rate of any diagnosis per 1,000 person-years (95% CI) | 790.5  (786.7-794.4) | 596.8  (593.6-600.1) | 1.36  (1.35 - 1.38) | 249.0  (246.8-251.3) | 259.0  (256.7-261.2) | 1.00  (0.98 - 1.02) | 82.6  (81.9 - 83.3) | 80.3  (79.6 - 80.9) | 1.06  (1.05 - 1.08) |
| **PVD** |  |  |  |  |  |  |  |  |  |
| Events (person-years) ^c^ | 89670 (222105) | 71367 (224996) |  | 31368 (206959) | 27015 (212280) |  | 43269 (782286) | 38725 (811071) |  |
| Incidence rate of any diagnosis per 1,000 person-years (95% CI) | 403.7  (401.1-406.4) | 317.2  (314.9-319.5) | 1.23  (1.21 - 1.24) | 151.6  (149.9-153.3) | 127.3  (125.8-128.8) | 1.18  (1.15 - 1.21) | 55.3  (54.8 - 55.8) | 47.7  (47.3 - 48.2) | 1.16  (1.14 - 1.18) |
| **Cerebrovascular outcomes** |  |  |  |  |  |  |  |  |  |
| **All Stroke** |  |  |  |  |  |  |  |  |  |
| Events (person-years) ^c^ | 2391 (234695) | 2185 (234976) |  | 2203 (231177) | 2178 (231605) |  | 8103 (924742) | 8086 (925795) |  |
| Incidence rate of any diagnosis per 1,000 person-years (95% CI) | 10.2  (9.8 -10.6) | 9.3  (8.9 - 9.7) | 1.06  (0.97 - 1.15) | 9.5  (9.1 - 9.9) | 9.4  (9.0 - 9.8) | 0.98  (0.90 - 1.06) | 8.8  (8.6 - 9.0) | 8.7  (8.5 - 8.9) | 0.97  (0.93 - 1.02) |
| **AIS** |  |  |  |  |  |  |  |  |  |
| Events (person-years) ^c^ | 1439 (234805) | 1432 (235053) |  | 1401 (231446) | 1396 (231814) |  | 5188 (927501) | 5223 (928233) |  |
| Incidence rate of any diagnosis per 1,000 person-years (95% CI) | 6.1  (5.8 - 6.5) | 6.1  (5.8 - 6.4) | 0.97  (0.87 - 1.07) | 6.1  (5.7 - 6.4) | 6.0  (5.7 - 6.3) | 0.97  (0.87 - 1.07) | 5.6  (5.4 - 5.7) | 5.6  (5.5 - 5.8) | 0.96  (0.91 - 1.01) |
| **Hemorrhagic Stroke** |  |  |  |  |  |  |  |  |  |
| Events (person-years) ^c^ | 222 (234950) | 212 (235185) |  | 227 (231795) | 243 (232152) |  | 884 (931336) | 894 (931983) |  |
| Incidence rate of any diagnosis per 1,000 person-years (95% CI) | 0.9  (0.8 - 1.1) | 0.9  (0.8 - 1.0) | 1.01  (0.77 - 1.31) | 1.0  (0.9 - 1.1) | 1.0  (0.9 - 1.2) | 0.90  (0.70 - 1.16) | 0.9  (0.9 - 1.0) | 1.0  (0.9 - 1.0) | 0.95  (0.84 - 1.09) |
| **TIA** |  |  |  |  |  |  |  |  |  |
| Events (person-years) ^c^ | 5369 (234218) | 3944 (234696) |  | 4002 (230220) | 3465 (230971) |  | 13351 (915471) | 11492 (919253) |  |
| Incidence rate of any diagnosis per 1,000 person-years (95% CI) | 22.9  (22.3 - 23.5) | 16.8  (16.3 - 17.3) | 1.34  (1.27 - 1.42) | 17.4  (16.9 - 17.9) | 15.0  (14.5 - 15.5) | 1.14  (1.07 - 1.22) | 14.6  (14.3 - 14.8) | 12.5  (12.3 - 12.7) | 1.16  (1.12 - 1.20) |

Abbreviations: AFIB, Atrial Fibrillation; AIS, Acute Ischemic Stroke; AMI, Acute Myocardial Infarction; CI, confidence interval; DVT, Deep Vein Thrombosis; HF, Heart Failure; HR, hazard ratio; IHD, Ischemic Heart Disease; PE, Pulmonary Embolism; PVD, Peripheral Vascular Disease; TIA, Transient Ischemic Attack.

^a^ For prevalence rates and HRs, the analyses included full cohort of FFS beneficiaries with non-hospitalized COVID-19 and matched controls.

^b^ In addition to propensity score matching, HRs were adjusted for all matching variables to control for potential residual confounding. 95% CIs were Bonferroni corrected 95% CI.

^c^ Prevalent events and person-years were calculated separately for 0-3, 4-6 and ≥7-month follow-up.
