# Supplementary material for: Long-term cardiovascular disease outcomes in non-hospitalized medicare beneficiaries diagnosed with COVID-19: Population-based matched cohort study
Source: PLoS One. 2024 May 14;19(5):e0302593. doi: 10.1371/journal.pone.0302593 (PMC11093379; doi:10.1371/journal.pone.0302593)
Supplement: Attachment — Submitted filename: PlosOne-ResponseToReviewers_0326-2024.docx [file pone.0302593.s009.docx]

PONE-D-23-36042: Long-Term Cardiovascular Disease Outcomes in Non-Hospitalized Medicare Beneficiaries Diagnosed with COVID-19: Population-Based Matched Cohort Study

**Response to Editor’s comments**

1. Please ensure that your manuscript meets PLOS ONE's style requirements, including those for file naming. The PLOS ONE style templates can be found at…

Response: we have followed the PLOS ONE’s style requirements in the revised version.

2. We note that you have indicated that there are restrictions to data sharing for this study. For studies involving human research participant data or other sensitive data, we encourage authors to share de-identified or anonymized data. However, when data cannot be publicly shared for ethical reasons, we allow authors to make their data sets available upon request. For information on unacceptable data access restrictions, please see <http://journals.plos.org/plosone/s/data-availability#loc-unacceptable-data-access-restrictions>.

Response: The Medicare data we have access to is the de-identified data, we can’t share these data because of the Data Use Agreement with the Centers for Medicare and Medicaid Services (CMS). Investigators can submit an application to CMS to access the Medicare data.

Before we proceed with your manuscript, please address the following prompts:

a) If there are ethical or legal restrictions on sharing a de-identified data set, please explain them in detail (e.g., data contain potentially identifying or sensitive patient information, data are owned by a third-party organization, etc.) and who has imposed them (e.g., a Research Ethics Committee or Institutional Review Board, etc.). Please also provide contact information for a data access committee, ethics committee, or other institutional body to which data requests may be sent.

Response: At the end of Methods section, we revised the data availability statement as follows:

**Data Availability:** The Medicare beneficiaries’ data used in this study to generate the findings are not publicly available and the authors cannot share these data due to the Data Use Agreement with the Centers for Medicare and Medicaid Services (CMS). Medicare data are available from CMS following a data use request from the third-party vendor, the Research Data Assistance Center (ResDAC). For instructions, guidance, and costs of CMS data, contact the ResDAC (<http://www.resdac.org>).

b) If there are no restrictions, please upload the minimal anonymized data set necessary to replicate your study findings to a stable, public repository and provide us with the relevant URLs, DOIs, or accession numbers. Please see <http://www.bmj.com/content/340/bmj.c181.long> for guidelines on how to de-identify and prepare clinical data for publication. For a list of recommended repositories, please see <https://journals.plos.org/plosone/s/recommended-repositories>. You also have the option of uploading the data as Supporting Information files, but we would recommend depositing data directly to a data repository if possible.

Please update your Data Availability statement in the submission form accordingly.

Response: As described above, we can’t share the Medicare beneficiaries’ data because of the Data Use Agreement with CMS. We have updated our Data Availability statement.

3. Please include captions for your Supporting Information files at the end of your manuscript, and update any in-text citations to match accordingly. Please see our Supporting Information guidelines for more information: http://journals.plos.org/plosone/s/supporting-information.

Response: We have included the tables and figure captions for supporting information files and we have updated the in-text citations accordingly in the revised version.

4. Please review your reference list to ensure that it is complete and correct. If you have cited papers that have been retracted, please include the rationale for doing so in the manuscript text, or remove these references and replace them with relevant current references. Any changes to the reference list should be mentioned in the rebuttal letter that accompanies your revised manuscript. If you need to cite a retracted article, indicate the article’s retracted status in the References list and also include a citation and full reference for the retraction notice.

Response: We have checked the references and reformatted the citations to PLOS ONE style. Please let me know if we may need to make any additional changes in the reference style.

**Academic Editor’s Comments:**

I commend the authors on the application of rigorous standard statistical analysis.
This is a noteworthy subject, which was dealt with in a structured and skillful manner.

Response: Thank you for your time to review the manuscript and providing thoughtful and constructive comments and suggestions.

1. Please provide a definition of this ICD-10 code on first use: “B97.29”

Response: We have added the definition for ICD-10 B97.29: “Other coronavirus as the cause of diseases classified elsewhere”

2. 161: Please provide the URL as a reference or place it in the supplementary file.

Response: We have changed the URL as a reference in the revised manuscript.

3. 321: Please delete the URL in the parenthesis.

Response: We have deleted this URL in the revised version.

4. 359: Please delete the URL in the parenthesis and a provide proper citation.

Response: We have deleted this URL and provided a citation.

5. Please discuss the probable mechanisms underlying the observed time-varying effect of SARS-CoV-2 infection on the CVD outcomes.

Response: Thank you for this thoughtful comment. Few COVID-19 and CVD studies have examined the time-varying effects of COVID-19 on risk of CVD and stroke. The probable mechanisms underlying the observed time-varying effects of SARS-CoV-2 infection on the CVD outcomes are unclear. In the Discussion, we have added: “A recent study showed that SARS-CoV-2 directly infects the coronary vessels and induces atherosclerotic plaque inflammation. These changes could trigger acute cardiovascular complications and increase the risk of CVD.[26] The direct effect of COVID-19 on coronary vessels and plaque inflammation, especially among those who had cardiovascular risk factors such as hypertension, diabetes, older age, or with preexisting CVD or stroke,[2, 4, 27] may help to explain the initial stronger effects of COVID-19 on CVD observed in the present study. However, COVID-19 can affect multiple organ systems and has numerous persistent symptoms, including long-term complications and increased risk of CVD and stroke (beyond 4 weeks from the onset of symptoms).[2, 4] There are several other proposed mechanisms by which COVID-19 could be associated with increased risk of CVD and stroke. COVID-19 may have indirect effects through systemic inflammatory responses to the infection or cytokine storm that could lead to cell death and multiorgan dysfunctions or through the hypoxia-induced myocardial injury due to hypoxic respiratory failure and hypoxemia, or small vessel ischemia due to microvascular injury and thrombosis.[2, 4, 27-29] Further studies are needed to understand the underlying mechanisms of acute and long COVID-19 and its cardiovascular sequelae.”

Reviewer #1: Good timely and needy work for the current phase.

Response: Thank you for reviewing the manuscript.

1. Do all the confounding factors have been analyzed and excluded.

Response: We have included a comprehensive list of demographic, socioeconomic and comorbidity conditions in the propensity score matching. The matching variables and potential confounding factors were from seven domains: Demographic factors; Socio-economic conditions; Healthcare utilization characteristics; Frailty characteristics; 37 Chronic Condition Warehouse (CCW) Conditions; CMS Hierarchical Condition Category (CMS-HCC) risk score; and Charlson Comorbidity Index. We have checked each variable before matching and excluded the incomplete potential confounders (excluded confounders are not shown in S1 Table).

2. Is there any strain specificity with the CV outcomes of the patients in long run follow up.

Response: Thank you for the thoughtful comments. Our baseline cohort included the early phase COVID-19 patients from April 1, 2020 to April 30, 2021 before the Delta variant predominance that started in July 15, 2021. For early phase of COVID-19, there is no detailed information of COVID-19 variants in Medicare data. We have added a sentence to the limitations indicating that we were unable to stratify our analyses by strain of COVID-19.

Reviewer #2: Findings of this study is of great value and fulfill gaps existing in literature. Statistical analysis is rigorous and support presented data. I don’t have any additional comments or suggestions to improve the text of the articles. Great job!

Response: We appreciate that the reviewer sees the value of our study, and we are grateful for your review of our manuscript.

Reviewer #3: it’s a great pleasure to read this new area of interest topic.

Response: Thank you for your time to review the manuscript.

1. Is there any strain specifically noted for the CV morbidity and mortality?

Response: Thank you for the thoughtful comments. Our baseline cohort included the early phase COVID-19 patients from April 1, 2020 to April 30, 2021 before the Delta variant predominance that started in July 15, 2021. For early phase of COVID-19, there is no detailed information of COVID-19 variants in Medicare data. We have added a sentence to the limitations indicating that we were unable to stratify our analyses by strain of COVID-19.

2. Is there any data regarding worsening of previous CV patients?

Response: In the sensitivity analysis, we compared the incidence rates and adjusted hazard ratio for risk of CVD and stroke associated with COVID-19 by follow-up time by excluding pre-existing CVD and stroke vs. including pre-existing CVD and stroke; the pattern of association remained largely consistent. Here are a few selected conditions (in the table below), please see supporting information S4 Table and S2 Figure for more detailed results.

Comparison of incidence rates and adjusted hazard ratio (95% CI) for risk of CVD and stroke associated with non-hospitalized COVID-19 by follow-up time: excluding pre-existing CVD and stroke vs. including pre-existing CVD and stroke), Medicare 2020–2021 Matched Cohort

|  | **0-3 month** | | | **4-6 month** | | | **≥7 month** | | |
| --- | --- | --- | --- | --- | --- | --- | --- | --- | --- |
|  | **COVID-19 status** | | | **COVID-19 status** | | | **COVID-19 status** | | |
| **Conditions** | **Yes** | **No** | **HR (95% CI)** ^b^ | **Yes** | **No** | **HR (95% CI)** ^b^ | **Yes** | **No** | **HR (95% CI)** ^b^ |
| **AMI** |  |  |  |  |  |  |  |  |  |
| Excluding pre-existing conditions |  |  |  |  |  |  |  |  |  |
| Events (person-years) **^c^** | 1,948 (222,974) | 1,622 (224,084) |  | 1,777 (219,922) | 1,685 (221,135) |  | 6,722 (882,462) | 6,708 (886,647) |  |
| Incidence rate per 1,000 person-years (95% CI) | 8.7  (8.4 - 9.1) | 7.2  (6.9 - 7.6) | 1.18  (1.07 - 1.29) | 8.1  (7.7 - 8.5) | 7.6  (7.3 - 8.0) | 1.04  (0.94 - 1.14) | 7.6  (7.4 - 7.8) | 7.6  (7.4 - 7.7) | 0.99  (0.94 - 1.03) |
| Including pre-existing conditions |  |  |  |  |  |  |  |  |  |
| Events (person-years) ^c^ | 2424 (234690) | 2054 (234987) |  | 2205 (231172) | 2079 (231619) |  | 8119 (924783) | 7980 (926034) |  |
| Incidence rate of any diagnosis per 1,000 person-years (95% CI) | 10.3  (9.9 - 10.7) | 8.7  (8.4 - 9.1) | 1.14  (1.05 - 1.23) | 9.5  (9.1 - 9.9) | 9.0  (8.6 - 9.4) | 1.02  (0.94 - 1.11) | 8.8  (8.6 - 9.0) | 8.6  (8.4 - 8.8) | 0.98  (0.94 - 1.03) |
| **AFIB and Flutter** |  |  |  |  |  |  |  |  |  |
| Excluding pre-existing conditions |  |  |  |  |  |  |  |  |  |
| Events (person-years) **^c^** | 9,616 (189754) | 5,725 (192,599) |  | 5,785 (186,578) | 5,556 (189,669) |  | 1,9626 (742,676) | 18,760 (755,169) |  |
| Incidence rate per 1,000 person-years (95% CI) | 50.7  (49.7 - 51.7) | 29.7  (29.0 - 30.5) | 1.69  (1.61 - 1.76) | 31.0  (30.2 - 31.8) | 29.3  (28.5 - 30.1) | 1.05  (1.00 - 1.10) | 26.4  (26.1 - 26.8) | 24.8  (24.5 - 25.2) | 1.05  (1.03 - 1.08) |
| Including pre-existing conditions |  |  |  |  |  |  |  |  |  |
| Events (person-years) ^c^ | 113373 (214844) | 94310 (220100) |  | 23326 (202591) | 26537 (207071) |  | 32303 (786817) | 33217 (801038) |  |
| Incidence rate of any diagnosis per 1,000 person-years (95% CI) | 527.7  (524.6-530.8) | 428.5  (425.8-431.2) | 1.31  (1.30 - 1.33) | 115.1  (113.7-116.6) | 128.2  (126.6-129.7) | 0.96  (0.93 - 0.98) | 41.1  (40.6 - 41.5) | 41.5  (41.0 - 41.9) | 1.02  (1.00 - 1.05) |
| **PE** |  |  |  |  |  |  |  |  |  |
| Excluding pre-existing conditions |  |  |  |  |  |  |  |  |  |
| Events (person-years) **^c^** | 1,919 (233580) | 732 (234,175) |  | 952 (230,294) | 805 (231,070) |  | 3397 (924,060) | 3110 (926,559) |  |
| Incidence rate per 1,000 person-years (95% CI) | 8.2  (7.9 - 8.6) | 3.1  (2.9 - 3.4) | 2.55  (2.26 - 2.87) | 4.1  (3.9 - 4.4) | 3.5 (  3.3 - 3.7) | 1.15  (1.01 - 1.31) | 3.7  (3.6 - 3.8) | 3.4 (  3.2 - 3.5) | 1.06  (0.99 - 1.14) |
| Including pre-existing conditions |  |  |  |  |  |  |  |  |  |
| Events (person-years) ^c^ | 1985 (234690) | 755 (235130) |  | 978 (231358) | 832 (231990) |  | 3499 (928178) | 3190 (930128) |  |
| Incidence rate of any diagnosis per 1,000 person-years (95% CI) | 8.5  (8.1 - 8.8) | 3.2 (3.0 - 3.4) | 2.55  (2.26-2.87) | 4.2  (4.0 - 4.5) | 3.6  (3.4 - 3.8) | 1.14  (1.00 - 1.30) | 3.8  (3.6 - 3.9) | 3.4  (3.3 - 3.6) | 1.07  (1.00 - 1.14) |
| **HF** |  |  |  |  |  |  |  |  |  |
| Excluding pre-existing conditions |  |  |  |  |  |  |  |  |  |
| Events (person-years) **^c^** | 7,723 (174,171) | 4,818 (177,732) |  | 5,176 (171,706) | 4,524 (175,571) |  | 15,197 (685,180) | 14,997 (702,170) |  |
| Incidence rate per 1,000 person-years (95% CI) | 44.3  (43.4 - 45.3) | 27.1  (26.4 - 27.9) | 1.63  (1.55 - 1.71) | 30.1  (29.3 - 31.0) | 25.8  (25.0 - 26.5) | 1.17  (1.10 - 1.23) | 22.2  (21.8 - 22.5) | 21.4  (21.0 - 21.7) | 1.04  (1.01 - 1.07) |
| Including pre-existing conditions |  |  |  |  |  |  |  |  |  |
| Events (person-years) ^c^ | 78569 (221405) | 59936 (226068) |  | 20990 (211420) | 21654 (216181) |  | 34614 (822055) | 35185 (840370) |  |
| Incidence rate of any diagnosis per 1,000 person-years (95% CI) | 354.9  (352.4-357.4) | 265.1  (263.0-267.3) | 1.32  (1.30 - 1.34) | 99.3  (97.9 -100.6) | 100.2  (98.8 -101.5) | 0.98  (0.96 - 1.01) | 42.1  (41.7 - 42.6) | 41.9  (41.4 - 42.3) | 0.99  (0.97 - 1.01) |
